# Hot life in Antarctica: a novel metabolically versatile *Pyrodictiaceae* genus thriving at a volcanic–cryosphere–marine interface

**DOI:** 10.1093/ismeco/ycag080

**Published:** 2026-03-27

**Authors:** Ana Carolina de Araújo Butarelli, Francielli Vilela Peres, Vivian Helena Pellizari, Amanda Gonçalves Bendia

**Affiliations:** Department of Biological Oceanography, Oceanographic Institute, University of São Paulo. Praça do Oceanográfico, 191, São Paulo, SP 05508-900, Brazil; Department of Biological Oceanography, Oceanographic Institute, University of São Paulo. Praça do Oceanográfico, 191, São Paulo, SP 05508-900, Brazil; Department of Biological Oceanography, Oceanographic Institute, University of São Paulo. Praça do Oceanográfico, 191, São Paulo, SP 05508-900, Brazil; Department of Biological Oceanography, Oceanographic Institute, University of São Paulo. Praça do Oceanográfico, 191, São Paulo, SP 05508-900, Brazil

**Keywords:** hyperthermophiles, archaea, metagenome, Antarctica, volcano

## Abstract

Deception Island fumaroles in Antarctica represent rare environments where extreme heat intersects with cryospheric and marine conditions, creating remarkable environmental gradients. From the near-boiling sediments, we reconstructed a high-quality metagenome-assembled genome affiliated with the *Pyrodictiaceae*. Phylogenomic analyses revealed that this genome, proposed to represent Ca. Pyroantarcticum pellizari, forms a distinct lineage separated from known genera in the family. Functional annotation uncovered a versatile metabolic repertoire, including pathways for sulfur and nitrogen cycling, peptide and amino acid transport, and mixotrophic energy conservation. Stress-response systems such as reverse gyrase, thermosome, and small heat-shock proteins were complemented by lineage-specific genes related to membrane stability, metal detoxification, and *Pyrodictiaceae*-specific cannulae. These adaptations likely support survival under sharp temperature gradients, hydrogen sulfide emissions, and high metal concentrations at the volcanic–cryosphere–marine interface. Our findings expand the phylogenetic and ecological scope of *Pyrodictiaceae*, highlighting Antarctic marine volcanoes as unique refuges for hyperthermophiles and as valuable models for investigating life’s habitability under extreme temperatures.

## Graphical Abstract

**Graphical Abstract:**
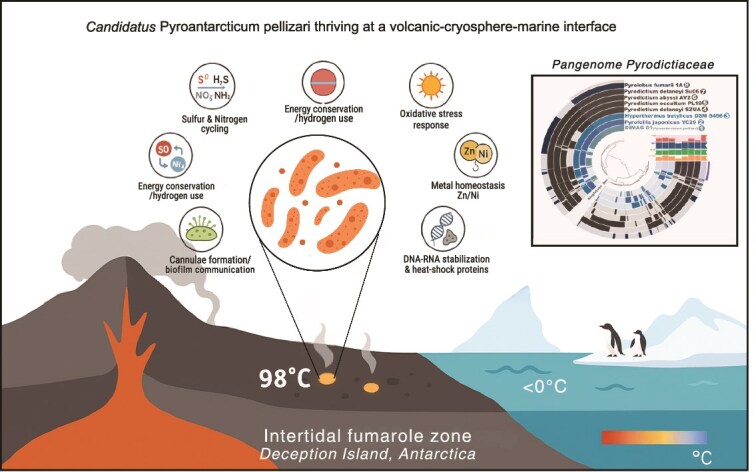

## Introduction

Hyperthermophilic Archaea, thriving at temperatures exceeding 80°C, represent life’s remarkable adaptability to Earth’s most extreme environments. These organisms, predominantly in the phyla *Crenarchaeota* and *Euryarchaeota*, inhabit geothermal springs, deep-sea hydrothermal vents, and subsurface oil reservoirs, where they drive critical biogeochemical cycles through, e.g. sulfur reduction, hydrogen oxidation, and chemolithoautotrophy [1, 2]. Their unique molecular adaptations, thermostable proteins, DNA repair systems, and ether-linked membrane lipids enable survival under conditions that destabilize most biomolecules [3–5]. Among these extremophiles, members of the family *Pyrodictiaceae* have been found in shallow and deep-sea hydrothermal vents [1, 6–8] and are described as obligate anaerobes, relying on sulfur reduction and hydrogen metabolism to sustain energy production in high-temperature, sulfur-rich habitats [9]. They encompass genera such as *Pyrodictium*, *Hyperthermus*, and *Geogemma*. Their metabolic flexibility and phylogenetic positioning near the root of the archaeal tree suggest ancient evolutionary origins, offering clues to early life’s adaptation to extreme environments [10]. However, genomic studies of *Pyrodictiaceae* have been limited by challenges in cultivation and genome recovery from low-biomass samples. *Pyrodictiaceae* members stand out for their remarkable metabolic versatility, a trait that may underlie their success in colonizing contrasting thermal environments such as deep-sea hydrothermal vents and polar volcanoes [7, 11, 12].

*Pyrodictiaceae* was first detected in Antarctica [11], together with other hyperthermophilic Archaea and bacteria in fumarolic sediments of active volcanic systems such as Deception Island, where the elevated temperatures intersect with the cryosphere [13]. These environments are unique in combining geothermal activity with direct marine and cryospheric influences, creating niches distinct from those in non-polar hydrothermal systems [14]. The coexistence of extreme heat with adjacent subzero conditions poses strong selective pressures, likely favoring microbial lineages with physiological plasticity and stress-response mechanisms [15, 16]. Although the distribution of hyperthermophiles in Antarctica is limited by the spatial isolation of geo- and hydrothermal hotspots, their persistence underscores the resilience and dispersal potential of hyperthermophilic lineages in polar regions. Studying these extremophiles not only expands our understanding of microbial adaptation and survival strategies in extreme environments, but also provides valuable analogs for exploring life in extraterrestrial icy worlds where volcanism and cryo-environments may co-occur [13, 17].

In this study, we investigated the metabolic potential and survival strategies of a novel, uncultivated hyperthermophilic Archaea obtained from metagenomic data of an active volcano in Antarctica. Using shotgun metagenomics and metagenome-assembled genome (MAG) reconstruction from an active fumarole site, we focused on: (i) determine the phylogeny and taxonomic placement of the genome; (ii) described its functional repertoire, with emphasis on sulfur, nitrogen, and carbon metabolisms; (iii) explored molecular adaptations associated with survival under extreme thermal conditions; and (iv) performed comparative genomic analyses with reference genomes from the *Pyrodictiaceae* to gain insights into phylogenomic placement, genome evolution, and ecological niche specialization in polar volcanic environments.

## Materials and methods

### Sampling and *in situ* analysis

**Figure 1:**
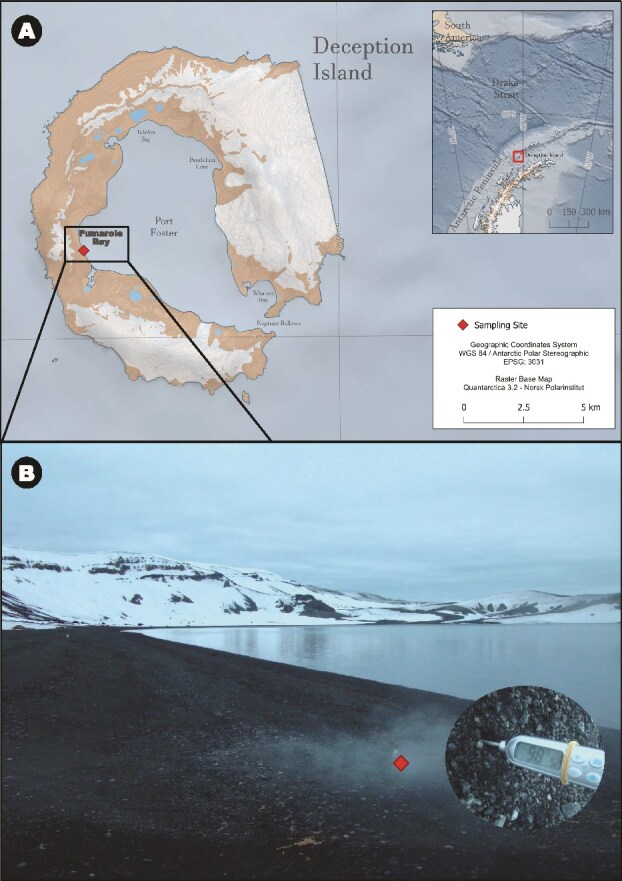
Geographic location and environmental setting of the fumarolic sampling site at Deception Island (Antarctica). (A) Geographic location of the sampling site at Deception Island, south Shetland Islands, Antarctica. The red diamond indicates the fumarolic vent at Fumarole Bay, situated on the inner coast of Port Foster. The map shows the position of Deception Island in relation to the Antarctic Peninsula (inset) and highlights the coastal fumarolic area. (B) Field view of the sampling site, where steaming fumarolic activity occurs directly at the shoreline. Three surface sediment samples at the vent were collected (samples FBA1, FBA2, and FBA3), with an *in situ* temperature of 98°C. The fumarolic deposits are characterized by dark volcanic sand with visible gas emissions, reflecting the strong hydrothermal influence on this coastal environment.

We collected three sediment samples (FBA1, FBA2, and FBA3) within a 15 cm radius in an active fumarole at Fumarole Bay, Deception Island volcano, Antarctica (62°58′02.7″ S, 60°42′ 36.4″ W) (Fig. 1). This fumarole was located in an intertidal zone, and the measured temperature was 98°C. More details about the sampling site are detailed in previous studies [11, 18]. We used a multiparametric probe (Horiba U-50) to measure *in situ* parameters in seawater near the fumarole: water temperature, pH, ORP (mV), conductivity (mS/cm), turbidity (NTU), OD (mg/L), OD (%), TDS (g/L), salinity (%), density (σt), and depth (m). We also performed physico-chemical parameters measurements of fumarole sediments [18], which included pH, temperature, granulometry (sand, silt, and clay), conductivity (EC), micronutrients (B, Cu, Fe, Mn, and Zn), organic matter (OM), organic carbon (OC), P, Si, Na, K, Ca, Mg, Al, total nitrogen, nitrate, ammonia, and sulfate. Samples were stored for DNA extraction at −20°C until arrival at the University of São Paulo, Brazil in April 2014.

### Deoxyribonucleic acid extraction and metagenomic sequencing

Total genomic DNA was extracted separately from 10 g of sediment from each of the three collected samples using the PowerMax Soil DNA Kit (MoBio, United States). The extracted DNA was then concentrated and purified using the OneStep PCR Inhibitor Removal Kit (Zymo Research, United States) [11, 18]. The three fumarolic samples were submitted to library constructions and to shotgun metagenomic sequencing, which were carried out at “Laboratório Central de Tecnologias de Alto Desempenho em Ciências da Vida” (LaCTAD), State University of Campinas (UNICAMP), on the Illumina Hiseq 2000 platform using 2x100 bp paired-end system.

### Binning and genome annotation

Reads from the three metagenomes were quality-trimmed using Sickle [19] with a Phred score of >30. A total of ~60 million trimmed reads were used for phylogenetic and functional profiling of the metagenome. Reads were co-assembled using MEGAHIT v. 1.0.2. [20] and contigs larger than 1000 bp were binned using CONCOCT [21] through Anvi’o v. 8 pipeline [22]. Bins were then manually refined using “anvi-refine”, and completeness and contamination were estimated using “anvi-summarize” and CheckM v1.0.7 [23]. We selected 1 MAG, which showed the highest quality for further analysis.

The selected MAG was annotated using PROKKA v1.14.5 [24], RAST v1.073 [25], the Kyoto Encyclopedia of Genes and Genomes (KEGG) automatic annotation server v2.1 [26], PATRIC [27], and BLAST v2.10.0 at the NCBI database [28], and was taxonomically classified based on genome phylogeny using GTDB-Tk v2.3.2 [29].

### Comparative genomics

Phylogenomic analysis was conducted using the Insert Genome Into SpeciesTree v2.2.0 tool (available on KBase), which constructs a species tree based on 49 universally conserved core genes defined by Clusters of Orthologous Groups (COGs) [30].

For the pangenome analysis, we employed the Anvi’o v8 pipeline to compare genomes and identify gene clusters. As our MAG with the highest quality was assigned to the *Pyrodictiaceae*, we selected publicly available genomes from this family (sourced from NCBI and IMG/JGI) for comparison. These included *Pyrofollis japonicus* YC29 (GCA_033097485.1), *Hyperthermus butylicus* DSM5456^**T**^ (GCA_000015145.1), *Pyrodictium delaneyi* SZUA (GCA_015661645.1), *Pyrodictium occultum* PL19 (GCA_001462395.1), *Pyrodictium abyssi* AV2 (GCA_036323395.1), *Pyrodictium delaneyi* Su06 (GCA_001412615.1), and *Pyrolobus fumarii* 1A (GCA_000223395.1). These reference genomes followed the same pipeline for functional annotation to enable direct comparison with our *Pyrodictiaceae* MAG*.*

To further assess genomic relatedness, we calculated Average Nucleotide Identity (ANI) and Average Amino Acid Identity (AAI) between our MAG and the reference genomes using EzANI and EzAAI, respectively, on our local server, applying default parameters (31). ANI calculations were performed using the EzANI pipeline [31], while AAI was computed with EzAAI [31, 32], and the calculation of the DDH distance was performed on the genome-to-genome website (GGDC) (http://ggdc.dsmz.de/home.php). The predicted proteins of strain DIMAG01 and selected reference genomes (*Pyrofollis japonicus* YC29, *H. butylicus* DSM5456^**T**^, *Pyrodictium delaneyi* SZUA, *P. occultum* PL19, *P. abyssi* AV2, *Pyrodictium delaneyi* Su06, *and P. fumarii* 1A) were submitted to the platform using default parameters [33]. The analysis provided information on the number of orthologous clusters, singletons, and shared gene families, which were used to assess the functional and evolutionary divergence among the compared genomes. The taxonomic assignment and nomenclature of the newly archaeal genome were carried out in accordance with the rules and recommendations of the SeqCode [34], which provides a standardized framework for the valid publication of prokaryotic names based on genome sequences serving as nomenclatural types.

## Results

### Co-assembly and recovery of the Pyrodictiaceae genome

By combining our three fumarole samples, we obtained a total of 50 193 556 filtered reads. The MAG reconstruction using three samples and a co-assembly approach yielded a 90.74% complete genome with 0.61% contamination, with a G + C content of 48.32% and a total genome size of ~1.46 Mb, which we named as DIMAG01. CheckM and GTDB-Tk assigned as the closest relative the genus *QNYQ01* in the *Pyrodictiaceae*, from the order Sulfolobales, phylum Thermoproteota. The annotation of the draft genome revealed a total of 1593 protein-coding genes. Among these, PROKKA classified 670 as proteins with functional assignments and 923 as hypothetical proteins. The most abundant Subsystem category classified by PATRIC was “Protein Processing” (90 proteins), followed by “Metabolism” (47 proteins), “Energy” (39 proteins), “RNA Processing” (24 proteins), “Membrane Transport” (15 proteins), “DNA Processing” (5 proteins), “Stress Response” (4 proteins), and “Cellular Processes” (1 protein). In the “Stress Response” category, proteins were classified as “Resistance to antibiotics and toxic compounds”, including copper tolerance (CutA), Hfl operon, integral membrane protein (MarC), and Glycerophosphoryl diester phosphodiesterase protein. KEGG annotation detected genes involved in assimilatory sulfate reduction (*sat*, K00958; *cysC*, K00860; *cysH*, K00390), nitrate reduction (*napA*, K02567; *napB*, K02568), and nitric oxide reduction (*norB*, K04561; *norC*, K02305). In addition, a gene annotated as *hao* (K10535) was detected. While *hao* is associated with nitrification in bacteria, its functional role in Archaea remains unclear. We also detected genes associated with oxidative stress response and heat-shock response, such as superoxide reductase (EC 1.15.1.2), thermosome (*thsA*, arCOG01257), reverse gyrase (arCOG01526), and the hyperthermophilic small heat-shock protein *hsp*16.5 (COG0071).

Recovery of rRNA genes was poor, which is not unusual for MAGs [35, 36]. Only the 23S and 5S rRNA genes were recovered, whose nucleotide sequences showed 96.6% identity to the corresponding rRNA genes of *H. butylicus* and *Pyrodictium delaneyi*. In addition, the *rpoB* gene and 29 tRNA genes were detected, with predicted anticodons for 15 amino acids. Based on *rpoB* nucleotide sequence comparison, the closest relative was *Pyrodictium delaneyi*, with 82% identity and 99% query coverage.

### Comparative genomics between metagenome-assembled genomes of the Pyrodictiaceae

The draft genome DIMAG01 (1.46 Mb, 60 contigs) is 90.7% complete, with a coding potential of 1134 CDS (Table 1). Values for the nearly complete reference genomes analyzed were 1.99 Mb, 1581 CDS, and 98.6% completeness for *Pyrofollis japonicus*, and 1.84 Mb, 1683 CDS, and 99.0% completeness for *P. fumarii* (Table 1). In terms of GC content, DIMAG01 (48.3%) is closer to *P. japonicus* (49.4%) and *H. butylicus* (53.7%) than to *P. abyssi* (63.5%) or *P. delaneyi* Su06 (59.2%) (Table 1). These comparisons suggest that DIMAG01 retains the general genomic features of *Pyrodictiaceae* (Table 1), though with a streamlined genome and slightly lower assembly completeness relative to the high-quality references.

**Table 1:** General genomic features of DIMAG01 and reference hyperthermophilic Archaea in the *Pyrodictiaceae*. Assembly statistics, genome quality (completeness and contamination), GC content, estimated chromosome size, and number of predicted protein-coding genes (CDS) are shown for DIMAG01 in comparison with representative reference genomes.

Features	Chromosome							
Taxonomy	Ca. Pyroantarcticum pellizari DIMAG01	*Pyrofollis japonicus* YC29^**T**^	*Hyperthermus butylicus* DSM 5456^**T**^	*Pyrodictium delaneyi* SZUA	*Pyrodictium occultum* PL19	*Pyrodictium abyssi* AV2	*Pyrodictium delaneyi* Su06	*Pyrolobus fumarii* 1A
GCA Accession	GCA_013540595.1	GCA_033097485.1	GCA_000015145.1	GCA_015661645.1	GCA_001462395.1	GCA_036323395.1	GCA_001412615.1	GCA_000223395.1
Source/environment	MAG assembled from Deception Island Metagenomes	Culture from deep-sea vent	Type strain of *Hyperthermus butylicus*	Hydrothermal vent metagenome	Culture from marine high temperature sediment	Hot abyssal vents (active smoker chimneys)	Culture from active hydrothermal chimney	From black smoker wall
Number of contigs	60	1	1	107	2	1	1	1
Completeness (%)	90.74	98.58	98.7	73.63	98.1	98.73	99.37	99.05
Contamination (%)	0.61	2.58	1.42	1.03	0.63	3.16	3.8	0.79
GC content (%)	48.32	49.36	53.74	52.93	63.5	59.17	53.91	54.9
Estimated chromosome size (bp)	1 457 804	2 005 174	1 667 163	879 958	1 621 727	2 224 219	2 023 836	1 843 267
Protein coding genes (CDS)	1134	1581	1473	797	1622	2154	1739	1683

The comparative analysis of overall genome-relatedness indices (Table 2) shows the genomic similarity metrics between *Pyrodictiaceae* sp. strain DIMAG01 and publicly available reference genomes in the *Pyrodictiaceae*, based on DNA–DNA hybridization (DDH), Average Nucleotide Identity (ANI), and AAI (Table 2). DDH values ranged from 20.6% (*H. butylicus* DSM5456^**T**^) to 32.6% (*P. occultum* strain PL-19), all well below the 70% threshold typically used to delineate species boundaries. Similarly, ANI values varied between 66.7% and 67.4%, below the ≥95% threshold indicative of species-level relatedness (Table 2). AAI values were relatively consistent across comparisons, ranging from 58.2% to 64.34%, reflecting low protein-level identity. These results collectively suggest that DIMAG01 represents a phylogenetically distinct lineage within the *Pyrodictiaceae* and support its proposal as a novel genus and species of hyperthermophilic Archaea (Table 2). Given the genomic distinctiveness, we propose that the MAG assembled from the Deception Island fumarole represents a novel species in a new genus of the *Pyrodictiaceae*, for which the name *Candidatus* Pyroantarcticum pellizari gen. nov., sp. nov. is proposed, in accordance with SeqCode rules and recommendations [34]. The genus name *Pyroantarcticum* highlights the hyperthermophilic nature of the lineage (*pyro-*, “fire”) and its origin in Antarctic geothermal systems, while the specific epithet *pellizari* honors the distinguished Brazilian microbiologist Vivian Pellizari, in recognition of her pioneering contributions to Antarctic microbial ecology.

**Table 2:** DDH, ANI, and AAI results utilizing *Pyrodictiaceae* DIMAG01 with query sequence.

GCA accession	Reference genome	DDH (%)	ANI (%)	AAI (%)
*GCA_000015145.1*	*Hyperthermus butylicus* DSM5456^**T**^	20.6	67.4	63.43
*GCA_001412615.1*	*Pyrodictium delaneyi* Su06	22.6	67.2	63.47
*GCA_001462395.1*	*Pyrodictium occultum* PL19	32.6	66.7	63.85
*GCA_036323395.1*	*Pirodictium abyssi* AV2	24.20	68.0	63.77
*GCA_015661645.1*	*Pyrodictium delaney* SZUA	24.80	68.1	64.34
*GCA_000223395.1*	*Pyrolobus fumarii* 1A	22.40	67.1	58.64
*GCA_033097485.1*	*Pyrofollis japonicus YC29* ^**T**^	21.40	67.5	62.65

In accordance with current SeqCode guidelines for genome-based taxonomy, the taxonomic assignment of strain DIMAG01 is supported primarily by genome-wide comparative analyses rather than by a single phylogenetic marker. Although a full-length 16S rRNA gene was not recovered, a recognized limitation of MAGs, taxonomic placement is robustly supported by phylogenomic reconstruction based on concatenated conserved marker genes, *rpoB* sequence comparisons, and multiple independent genome relatedness indices (ANI, AAI, and DDH). Together, these complementary lines of evidence provide a consistent and reliable framework for the genome-based delineation of a novel genus and species within the *Pyrodictiaceae*.

**Figure 2:**
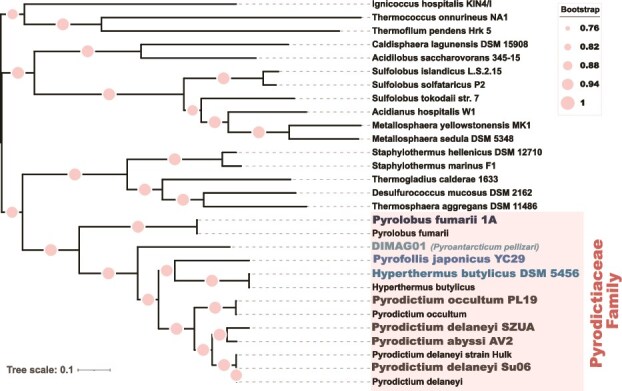
Phylogenomic tree showing the evolutionary relationships among representative members of the *Pyrodictiaceae*. The tree was constructed based on concatenated alignments of conserved single-copy marker genes. Bootstrap support values are indicated by circles, with larger and darker symbols representing higher confidence levels. Reference genomes belonging to the *Pyrodictiaceae* are shown in bold, and the family-level clade is indicated on the right. The tree scale bar represents 0.1 substitutions per site.

The phylogenomic analysis (Fig. 2) revealed that strain DIMAG01 forms a well-supported distinct clade in the *Pyrodictiaceae*, separated from extant genera such as genera such as *Pyrodictium*, *Hyperthermus*, and *Pyrolobus*. Its closest relative in the tree was *Pyrofollis japonicus* YC29^**T**^, yet the moderate bootstrap value and long branch length indicate significant evolutionary divergence (Fig. 2). The phylogenomic structure revealed that while DIMAG01 is affiliated with the *Pyrodictiaceae*, it does not cluster in an extant genus, highlighting its uniqueness.

This intermediate placement suggests that DIMAG01 may represent a transitional lineage in *Pyrodictiaceae*, retaining ancestral features or bridging evolutionary gaps between existing genera. The distinctiveness of this lineage is further supported by high bootstrap values, underscoring the stability and robustness of its phylogenetic position. Combined with the consistently low ANI, AAI, and DDH values observed in comparisons with available *Pyrodictiaceae* genomes (Table 2), these results strongly support the classification of DIMAG01 as a representative of a novel genus and species. Its discovery broadens the phylogenetic and ecological diversity of *Pyrodictiaceae* and provides new insights into Archaea lineages inhabiting Antarctic volcanic environments.

### Core genome—Pyrodictiaceae

**Figure 3:**
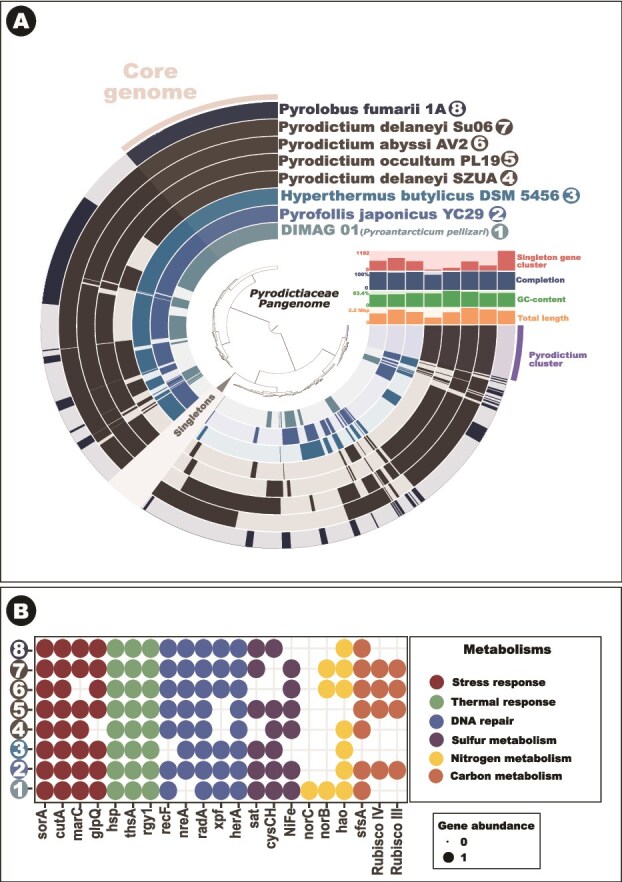
Comparative phylogenomics and functional profiling of the *Pyrodictiaceae* pangenome. (A) Pangenomic analysis of *Pyrodictiaceae* genomes using anvi’o v8. The concentric rings represent genomes included in the analysis, with each block corresponding to a gene cluster. The inset heatmap shows genome-specific metrics, including the proportion of singleton gene clusters, completeness, GC content, and total length (B) presence/absence matrix of functional genes involved in key metabolic pathways across representative *Pyrodictiaceae* genomes. Each colored dot indicates detection of genes associated with a specific functional category, including sulfur metabolism, stress and thermal response, DNA repair, and carbon, nitrogen, and sulfur metabolism.

The core genome analysis of the *Pyrodictiaceae* (Fig. 3a) revealed a compact yet functionally diverse set of conserved genes essential for survival in hyperthermophilic and extreme environments (Fig. 3). Key functional categories included RNA modification, ribosome biogenesis, protein quality control, cofactor and vitamin biosynthesis, membrane lipid metabolism, and fundamental replication and transcription machinery (Fig. 3b).

Post-transcriptional RNA modifications were prominently represented by methyltransferases such as 16S rRNA (cytosine967-C5)-methyltransferase (RsmB; EC 2.1.1.176), 16S rRNA (guanine1207-N2)-methyltransferase (RsmC; EC 2.1.1.172), and tRNA (guanine6-N2)-methyltransferase (Trm14; EC 2.1.1.256). The conserved tRNA (N6-threonylcarbamoyladenosine37-C2)-methylthiotransferase (TtcA; EC 2.8.4.5) highlights sulfur-relay pathways important for RNA stability under thermal stress.

Main metabolic pathways and representative genes identified in Ca. Pyroantarcticum pellizari are summarized in Table 3. Proteostasis-related genes included chaperonins such as heat shock protein 60 (HSP60; K04077), methionyl aminopeptidase (MetAP; EC 3.4.11.18; K01265), and components of the 20S proteasome complex (KO: K01332–K01334), indicating active protein folding and degradation mechanisms. Enzymes involved in nitrogen metabolism and redox homeostasis included glycine dehydrogenase (GcvP; EC 1.4.4.2; KO: K00301) and hydroxylamine dehydrogenase (Hao; EC 1.7.2.6; KO: K10535). Membrane bioenergetics relied on membrane-bound V/A-type H^+^/Na^+^-transporting ATPases (subunits A–E; KO: K02117–K02121) and archaeal membrane lipid biosynthesis enzymes such as sn-glycerol-1-phosphate dehydrogenase (G1PDH; EC 1.1.1.261; KO: K00075).

**Table 3:** Major metabolic pathways and representative genes identified in Ca. Pyroantarcticum pellizari.

Functional category/pathway	Representative genes (KEGG orthologs/EC numbers)
Proteostasis and protein quality control	HSP60 (K04077), methionyl aminopeptidase MetAP (EC 3.4.11.18; K01265), 20S proteasome subunits (K01332–K01334)
Nitrogen metabolism and redox homeostasis	Glycine dehydrogenase GcvP (EC 1.4.4.2; K00301), hydroxylamine dehydrogenase Hao (EC 1.7.2.6; K10535)
Membrane bioenergetics	V/A-type H^+^/Na^+^-transporting ATPase subunits A–E (K02117–K02121)
Archaeal membrane lipid biosynthesis	sn-glycerol-1-phosphate dehydrogenase G1PDH (EC 1.1.1.261; K00075)
Cofactor and vitamin biosynthesis	NadE (EC 6.3.1.5; K03740), NadD (EC 2.7.7.1; K00763), MetK (EC 2.5.1.6; K00789), HemC (EC 2.5.1.61; K01743), HemL (EC 5.4.3.8; K00838)
DNA replication and repair	DNA polymerase B PolB (K02323), MCM helicase (EC 5.6.2.4; K03631), DNA ligase LigA (EC 6.5.1.1; K01972), RecB/RecC (K03553, K03554), EndA (EC 3.1.21.3; K01142)
Transcription machinery	RNA polymerase subunits Rpo (K03043–K03047), transcription factors TFB (K03120) and TFE (K03119)
Regulatory systems	Ribbon–helix–helix transcriptional repressors, toxin–antitoxin systems MazEF (K19188), two-component systems (ko02020)
Amino acid metabolism	Cysteine and methionine metabolism (ko00270), arginine and proline metabolism (ko00330), glutathione metabolism (ko00480)
Methionine salvage pathway	Spermidine synthase SpeE (EC 2.5.1.16; K00797), FAD synthase Fads (EC 2.7.7.2; K03803)
Riboflavin metabolism	Flavin prenyltransferase (EC 2.5.1.129; K03743)
Aminoacyl-tRNA biosynthesis	AlaRS (K01875), AspRS (K01893), ArgRS (K01885), GlyRS (K01880), TyrRS (K01868), GlnRS (K01883)
Nucleotide metabolism	Pyrimidine metabolism: PyrB (EC 2.1.3.2; K00640), NrdA (EC 1.17.4.1; K00525); Purine metabolism (ko00230)
Central carbon metabolism	Glycolysis/gluconeogenesis: phosphoglycerate mutase Gpm (EC 5.4.2.12; K01834); serine/glycine/threonine metabolism (ko00260)
Pentose phosphate pathway	6-phospho-3-hexuloisomerase (EC 5.3.1.27; K06967)
Hydrolases	CysQ (EC 3.1.3.97; K01086), NTPase (EC 3.6.1.15; K01063)
Energy metabolism	Oxidative phosphorylation (ko00190)
Transport systems	ABC transporters (ko02010)
Oxidative stress response	Superoxide reductase SOR (EC 1.15.1.2; K00433)
Genetic information processing	Ribosomal proteins (ko03010), mRNA surveillance (ko03015)
Poorly characterized/unclassified genes	Poorly characterized genes (ko09194), unclassified metabolic genes (ko09191, ko09192)

Cofactor and vitamin biosynthesis genes included NAD^+^ synthase (NadE; EC 6.3.1.5; KO: K03740), nicotinamide-nucleotide adenylyltransferase (NadD; EC 2.7.7.1; KO: K00763), methionine adenosyltransferase (MetK; EC 2.5.1.6; KO: K00789), hydroxymethylbilane synthase (HemC; EC 2.5.1.61; KO: K01743), and glutamate-1-semialdehyde aminomutase (HemL; EC 5.4.3.8; KO: K00838), supporting porphyrin and heme biosynthesis. Replication and transcription machinery were complete and conserved, featuring DNA polymerase B (PolB; KO: K02323), replicative helicases (MCM; EC 5.6.2.4; KO: K03631), ATP-dependent DNA ligases (LigA; EC 6.5.1.1; KO: K01972), RNA polymerase subunits (Rpo; KO: K03043–K03047), and basal transcription factors TFB (KO: K03120) and TFE (KO: K03119). Regulatory elements included ribbon-helix–helix transcriptional repressors and toxin-antitoxin systems such as MazEF (KO: K19188).

The data highlighted amino acid metabolism genes involved in cysteine and methionine metabolism (ko00270), arginine and proline metabolism (ko00330), and glutathione metabolism (ko00480). The methionine salvage pathway included spermidine synthase (SpeE; EC 2.5.1.16; KO: K00797) and FAD synthase (Fads; EC 2.7.7.2; KO: K03803). Riboflavin metabolism (KO: K00740) enzymes such as flavin prenyltransferase (EC 2.5.1.129; KO: K03743) were also detected.

The aminoacyl-tRNA biosynthesis pathway (KO: K00970) included alanine–tRNA ligase (AlaRS; EC 6.1.1.7; KO: K01875), aspartate–tRNAAsn ligase (AspRS; EC 6.1.1.23; KO: K01893), arginine–tRNA ligase (ArgRS; EC 6.1.1.19; KO: K01885), glycine–tRNA ligase (GlyRS; EC 6.1.1.14; KO: K01880), tyrosine–tRNA ligase (TyrRS; EC 6.1.1.1; KO: K01868), and glutaminyl–tRNA synthase (GlnRS; EC 6.3.5.7; KO: K01883). Nucleotide metabolism pathways included pyrimidine metabolism enzymes such as aspartate carbamoyltransferase (PyrB; EC 2.1.3.2; KO: K00640) and ribonucleoside-diphosphate reductase (NrdA; EC 1.17.4.1; KO: K00525), purine metabolism (KO: K00230), and the pentose phosphate pathway enzyme 6-phospho-3-hexuloisomerase (EC 5.3.1.27; KO: K06967). Hydrolases such as 3′,5′-nucleoside bisphosphate phosphatase (CysQ; EC 3.1.3.97; KO: K01086) and nucleoside-triphosphate phosphatase (NTPase; EC 3.6.1.15; KO: K01063) were also present. Central carbon metabolism was supported by glycolysis/gluconeogenesis enzymes such as phosphoglycerate mutase (Gpm; EC 5.4.2.12; KO: K01834), and serine/glycine/threonine metabolism (KO: K00260).

Additional pathways included nicotinate and nicotinamide metabolism (ko00760), folate biosynthesis (ko00790) with GTP cyclohydrolase I (FolE; EC 3.5.4.16; KO: K01495), thiamine metabolism (ko00730), and riboflavin metabolism (ko00740). Genetic information processing included ribosomal proteins (ko03010), mRNA surveillance (ko03015), and DNA repair with RNA helicases (Ski2-like; EC 3.6.4.13; KO: K03688), homologous recombination RecBC pathway proteins (RecB and RecC; KO: K03553, K03554), and endonucleases (EndA; EC 3.1.21.3; KO: K01142). Energy metabolism featured oxidative phosphorylation (ko00190) and membrane transport systems such as ABC transporters (ko02010). Two-component regulatory systems (ko02020) and oxidative stress response enzymes like superoxide reductase (SOR; EC 1.15.1.2; KO: K00433) were also identified. A substantial fraction of genes remains poorly characterized or unclassified (KEGG: ko09194, ko09191, ko09192), reflecting current limitations in functional annotation and reference databases for hyperthermophilic archaea.

### Singleton genes of DIMAG01

Analysis of the singleton genes in the newly assembled *Pyrodictiaceae* genome (Fig. 3a) revealed a set of functionally diverse and taxonomically restricted elements that likely underpin this lineage’s unique adaptations to hyperthermophilic, anaerobic, and metal-rich environments. These singletons encode specialized enzymes, structural proteins, and transport systems not identified in other members of the *Pyrodictiaceae*, indicating lineage-specific innovations.

Several singletons are implicated in maintaining cellular integrity and functionality under extreme thermal stress. For example, the preflagellin peptidase FlaK (EC 3.4.23.52) processes archaellin precursors, facilitating the assembly of thermotolerant motility structures unique to Archaea (KEGG: archaeal flagellar system). Another critical component, the HerA DNA helicase (EC 3.6.4.13; KEGG ko03440), participates in homologous recombination and DNA repair at elevated temperatures, functioning alongside archaeal-specific repair complexes.

Singletons also encode enzymes supporting chemolithoautotrophic metabolism, particularly under anaerobic and sulfur-rich conditions. A hydrogenase maturation protease (*hyaD*) (EC 3.4.23) was identified, but no evidence for a complete, energy-conserving hydrogenase system was found. Similarly, the nitric oxide reductase *norB* (EC 1.7.2.5; KEGG: ko00910) enables anaerobic respiration via NO detoxification, a critical mechanism in vent-associated sulfur cycling.

In terms of cofactor biosynthesis, several singleton-encoded enzymes play roles in the assembly of thermally stable cofactors. The molybdopterin synthase complex (*moaE-moaD*) (EC 2.8.1.12; KEGG: ko00790, ko04122) facilitates biosynthesis of the molybdenum cofactor essential for redox enzymes such as formate dehydrogenases. Additionally, Cob(II)alamin adenosyltransferase (*pduO*) (EC 2.5.1.17; KEGG: ko00860) catalyzes the final step in the assembly of vitamin B₁₂, contributing to methyltransferase-dependent processes in thermophilic Archaea.

Thermostabilization of tRNA and RNA is supported by a suite of modification enzymes found uniquely in this genome. The tRNA G37 N-methylase (*Trm5*) (EC 2.1.1.) and pseudouridine 13 synthase (*TruD*) (EC 5.4.99.27; KEGG: ko03008) enhance RNA structural integrity through methylation and pseudouridylation, respectively, modifications known to prevent degradation under high-temperature conditions. Heavy metal transport and detoxification pathways also appear to be uniquely encoded by singletons. The nickel transporter permease complex (*dppB/dppC*) (KEGG: ko02010) facilitates uptake of Ni^2+^, a crucial cofactor for hydrogenases and ureases, while the zinc transporter ZupT (KEGG: ko04146) maintains Zn^2+^ homeostasis under sulfide-rich stress, a common feature of hydrothermal vent systems [37, 38].

Unique functions include stress response and quorum-sensing regulation. A quorum-quenching N-acyl-homoserine lactonase (EC 3.1.1.81; KEGG: ko02024) degrades signaling molecules used by other bacteria (domains Bacteria and Archaea), possibly conferring a competitive advantage in densely populated habitats. The Fe-S cluster assembly protein SufB (KEGG: ko04122) is likely involved in protecting essential redox centers from oxidative or thermal damage.

Crucially, a subset of singletons encodes *Pyrodictiaceae*-specific structures and functions. Several uncharacterized proteins with DUF58 and DUF447 domains, e.g. YeaD2, are likely associated with the formation of *cannulae*, a network of extracellular nanotubes exclusively observed in this family. Another distinctive feature is the presence of geranylgeranylglycerol-phosphate geranylgeranyltransferase (*ubiA*) (EC 2.5.1.42), responsible for the synthesis of thermostable archaeal membrane lipids, essential for maintaining membrane fluidity and integrity at high temperatures conditions.

Together, these lineage-specific genes underscore the functional innovations that enable *Pyrodictiaceae* DIMAG01 to thrive in diverse extreme environments, with a genome shaped by selective pressures favoring thermal resilience, metabolic versatility, and ecological competitiveness.

## Discussion

### Phylogenetic novelty and taxonomic implications

This study reports the first MAG of a hyperthermophilic archaea from Antarctica, strain DIMAG01, obtained from a high-temperature fumarole (98°C) at Deception Island. Phylogenomic analyses robustly place this genome in the Pyrodictiaceae, but consistently apart from known genera such as *Pyrodictium*, *Hyperthermus*, *Pyrolobus*, and *Pyrofollis*. In the species tree, DIMAG01 occupies a long, well-supported branch (bootstrap >90%), and pairwise comparisons show deep genomic divergence from reference genomes, with DDH (20.6%–32.6%), ANI (68.1%–66.7%), and AAI (58.64%–64.34%) values all falling well below genus-level thresholds.

Together, these data support the classification of DIMAG01 as representing a novel species in a novel genus in *Pyrodictiaceae*, for which we propose the provisional name “*Candidatus* Pyroantarcticum pellizari” gen. nov., sp. nov. Its recovery from a near-boiling (98°C) intertidal fumarole underscores the ecological singularity of Deception Island, where geothermal and polar conditions intersect to create unique habitat for extremophiles [11, 18]1). The phylogenetic distinctness of Ca. P. pellizari may reflect ancient divergence (Fig. 2), possible persistence of ancestral traits, or ecological specialization in the volcanism of the South Shetlands. These findings broaden the ecological and evolutionary boundaries of *Pyrodictiaceae* and point to Antarctica as a reservoir for a unique hyperthermophilic Archaea.

### Metabolic versatility under polar geothermal constraints

Functional genome Annotation reveals a metabolically flexible lifestyle finely tuned to the physicochemical conditions of Deception Island’s hydrothermal ecosystem (Supplementary Table 1 and 2).Ca. P. pellizari encodes genes associated with assimilatory sulfate reduction (*sat*, *cysC*, *cysH*) and nitrogen oxide transformations (*napAB*, *norBC*), suggesting sulfur assimilation and limited redox versatility in nitrogen metabolism. The detection of *hao*, involved in hydroxylamine oxidation, may represent an adaptive trait for nitrogen scavenging in this oligotrophic fumarolic niche. The presence of amino acid and peptide ABC transporters, and formate dehydrogenase supports a mixotrophic lifestyle, combining heterotrophy with limited chemolithotrophic capabilities, traits advantageous in chemically dynamic, substrate-limited volcanic sediments [39].

The metabolic profile of Ca. P. pellizari expands known capabilities of *Pyrodictiaceae*, which are typically sulfur-respiring and organotrophic [40]. The ability to process multiple electron donors and acceptors may reflect selection by fluctuating redox conditions in Deception Island fumarolic intertidal zones, which are subject to tidal exposure, continuous hydrogen sulfide emissions, and extreme geochemical/metal gradients [18, 41, 42].

### Molecular and structural adaptations to polar hyperthermophily

Comparative genomic analysis reveals both conserved and lineage-specific adaptations associated with extreme thermophily. Ca. P. pellizari retains canonical archaeal thermotolerance genes such as group II chaperonins (thermosome), small heat-shock proteins (*hsp16.5*), and reverse gyrase, critical for macromolecular stability at high temperatures. RNA and DNA protection mechanisms are reinforced by tRNA-modifying enzymes (Trm5, TruD, RsmB) and the archaeal helicase HerA, ensuring structural integrity of genetic material under thermal stress.

Remarkably, the genome encodes DUF58/DUF447 domain proteins, likely involved in the formation of cannulae, extracellular nanotube-like structures unique to *Pyrodictiaceae*, which enable cellular communication and nutrient exchange in dense biofilms [43, 44]. These structures may be critical for survival in sulphidic, metal-rich fumarolic sediments, and their presence supports retention of *Pyrodictiaceae*-defining phenotypes [43]. Additional adaptations include UbiA-mediated biosynthesis of archaeal membrane lipids (e.g. geranylgeranylglycerol phospholipids), nickel and zinc transporters (*dppBC*, *zupT*) for heavy metal homeostasis, and enzymes involved in cofactor biosynthesis (e.g. SufB, PduO). These features likely contribute to membrane stability, metal detoxification, and redox balancing in an environment characterized by high temperatures, reduced compounds due to hydrogen sulfide emissions, and trace metal abundance, such as those found in Deception Island fumaroles [45, 46].

Interestingly, Ca. P. pellizari also encodes oxidative stress response proteins (e.g. superoxide reductase) and quorum-quenching lactonases, traits uncommon in deep-sea *Pyrodictiaceae* but potentially advantageous in tidally influenced fumaroles where oxygen and microbial density may fluctuate drastically [18, 47, 48]. The presence of these genes suggests that, in addition to thermophily, this lineage has adapted to spatially and temporally heterogeneous conditions at the ocean-hydrothermal-cryosphere interface.

In contrast to deep-sea hydrothermal vent systems, which experience relatively stable physicochemical conditions, intertidal hydrothermal fumaroles are subject to rapid fluctuations in temperature, redox state, and oxygen availability driven by tidal mixing [49, 50]. Such environmental instability likely imposes strong selective pressure on cellular protection and regulatory systems. Accordingly, the presence of oxidative-stress response and signaling-related genes in Ca. P. pellizari may represent molecular adaptations that support cellular integrity and regulatory flexibility under recurrent thermal and redox stress, complementing structural strategies associated with polar hyperthermophily [51].

### Ecological and evolutionary insights from a polar hyperthermophile

The elevated number of singleton genes in Ca. P. pellizari, many involved in transport, respiration, stress response, and cofactor biosynthesis, may reflect genomic adaptation to its Antarctic marine volcanic habitat rather than these functions being unique per se. Its apparent phylogenetic distance from other known *Pyrodictiaceae* could result from allopatric diversification driven by the Southern Ocean biogeographic barriers [52], compounded by the rare combination of thermal and polar constraints shaping its evolutionary trajectory.

Deception Island represents a rare thermal refuge in the Southern Ocean [14, 53], and the discovery of Ca. P. pellizari highlights the potential for endemic archaeal lineages to arise under such unique environmental regimes. The capacity for sulfur and nitrogen cycling, together with structural features such as cannulae and stress-resistance systems, points to a survival strategy adapted to transient energy availability, metal stress, and community-level interactions in the sediments.

In this context, Ca. P. pellizari not only bridges evolutionary gaps in *Pyrodictiaceae* but also provides a valuable model to investigate how extremophilic life persists at the intersection of cold and heat. Its genome offers insights into the potential for microbial life to thrive in dual extremes, a theme increasingly relevant to studies in astrobiology, microbial bioprospecting, and the impacts of climate-driven change in polar ecosystems.

## Conclusion

This study reports a draft genome of a hyperthermophilic archaeon from Antarctica, expanding the ecological and evolutionary boundaries of the *Pyrodictiaceae*. The genome-centric analyses revealed a novel and metabolically versatile lineage, Ca. P. pellizari, obtained from near-boiling fumaroles on Deception Island. Its genetic repertoire highlights a combination of canonical hyperthermophilic traits and lineage-specific innovations, including sulfate reduction and denitrification pathways, stress-response systems, and structural adaptations that likely support survival under fluctuating thermal, geochemical, and cryospheric conditions. The discovery of this Archaea at the volcanic–cryosphere–marine interface demonstrates that Antarctic volcanoes harbor unique microbial lineages shaped by the interplay between heat and ice. These findings highlight the role of polar volcanoes as natural laboratories for studying microbial adaptation, dispersal, and evolution under multiple extremes, while also providing analogs for potential life strategies in extraterrestrial worlds where volcanism and cryo-environments coexist.

## Supplementary Material

Supplementary_material_DIMA01_ycag080

## Data Availability

The *Pyrodictiaceae* sp. DIMAG01 draft genome reported here is available in the DDBJ/EMBL/GenBank database under the accession number JABXKE000000000, Biosample SAMN14533095, BioProject PRJNA615910. The name “*Pyroantarcticum pellizari*” sp. nov. is proposed here in accordance with the SeqCode. The corresponding registration entry will become publicly available upon acceptance of the manuscript at the following link: https://seqco.de/i:52885.
