# Supplementary material for: Hot life in Antarctica: a novel metabolically versatile Pyrodictiaceae genus thriving at a volcanic–cryosphere–marine interface
Source: ISME Commun. 2026 Mar 27;6(1):ycag080. doi: 10.1093/ismeco/ycag080 (PMC13140500; doi:10.1093/ismeco/ycag080)
Supplement: Supplementary_material_DIMA01_ycag080 [file supplementary_material_dima01_ycag080.docx]

**Supplementary Table 1**. Weather conditions and superficial seawater properties recorded in Fumarole Bay (Deception Island, Antarctica). Atmospheric parameters include pressure, air temperature, wind speed and wind direction. Superficial seawater parameters were measured in situ using a multiparametric probe (Horiba U-50), comprising temperature, pH, oxidation-reduction potential (ORP), conductivity, turbidity, dissolved oxygen (DO), total dissolved solids (TDS), salinity, density, and sampling depth.

| ***Weather conditions on January 9, 2014 (~10 p.m.–1 a.m.) in Fumarole Bay*** | |
| --- | --- |
| *Atmospheric Pressure (hPa)* | 1004 |
| *Air Temperature (°C)* | -0,8 |
| *Wind speed (kt)* | 8 |
| *Wind speed (km/h)* | 15 |
| *Wind Direction* | 340° |
| ***Surface Water Properties in Fumarole Bay*** | |
| *Water Temperature (°C)* | -0,25 |
| *pH* | 4,97 |
| *ORP (mV)* | 147 |
| *Conductivity (mS/cm)* | 24,8 |
| *Turbidity (NTU)* | 0,4 |
| *DO (mg/L)* | 15,73 |
| *DO (%)* | 133 |
| *TDS (g/L)* | 15,4 |
| *Salinity (%)* | 2,98 |
| *Density (σt)* | 23,9 |
| *Depth (m)* | 0,1 |

**Supplementary Table 2.** Physicochemical parameters of fumarolic sediments collected at three sites (FBA1, FBA2 and FBA3) in Fumarole Bay (Deception Island, Antarctica). Measured variables include temperature, trace elements (B, Cu, Fe, Mn, Zn), organic matter and organic carbon, pH (H₂O and KCl), nutrients (P, Si, Na, K, Ca, Mg), base saturation (SB), cation exchange capacity (CEC), base saturation index (V), sulfate, total nitrogen, ammonium (NH₄⁺), nitrate (NO₃⁻), electrical conductivity, and sediment granulometry (sand, silt, clay)

|  | | ***FBA1*** | ***FBA2*** | ***FBA3*** |
| --- | --- | --- | --- | --- |
| ***Celsius*** | *Temperature (°C)* | 98 | 98 | 98 |
| ***mg.dm^-3^*** | *B* | 0,14 | 0,15 | 0,12 |
|  | *Cu* | 0,7 | 0,4 | 0 |
|  | *Fe* | 277 | 296 | 121 |
|  | *Mn* | 9,1 | 8 | 6,9 |
|  | *Zn* | 0,4 | 0,2 | 0,4 |
| ***g/kg*** | *Organic Matter* | 7 | 6 | 6 |
|  | *Organic Carbon* | 4 | 3 | 4 |
|  | *pH H2O* | 6,7 | 7 | 6,9 |
|  | *pH KCl* | 5,7 | 5,8 | 5,7 |
| ***(mg.Kg^-1^)*** | *P* | 42 | 49 | 40 |
|  | *Si* | 21 | 25 | 26 |
|  | *Na* | 15,1 | 19,4 | 21,5 |
|  | *K* | 3 | 3,9 | 3,3 |
|  | *Ca* | 7 | 7 | 8 |
|  | *Mg* | 10 | 12 | 10 |
| ***mmolc.Kg^-1^*** | *SB* | 34,8 | 42,5 | 42,5 |
|  | *CTC* | 37,4 | 45,1 | 46,8 |
| ***%*** | *V* | 93 | 94 | 91 |
|  | *m* | 1 | 1 | 0 |
| ***mg.dm^-3^*** | *Sulfate* | 125 | 118 | 92 |
|  | *N (total)* | 448 | 406 | 413 |
| ***mg/kg*** | *NH4+* | 70 | 35 | 39 |
|  | *NO-3* | 84 | 39 | 39 |
| ***μS/cm*** | *Eletric Condutivity* | 494 | 429 | 610 |
| ***g/kg*** | *Sand* | 941 | 939 | 924 |
|  | *Silt* | 22 | 11 | 26 |
|  | *Clay* | 37 | 50 | 50 |

**Supplementary Figure 1.** Environmental scanning electron microscopy (ESEM) image of fumarolic sediments collected at Fumarole Bay (Deception Island, Antarctica).Red arrows indicate filamentous extracellular structures that may correspond to cannulae-like networks, a hallmark of the Pyrodictiaceae lineage.


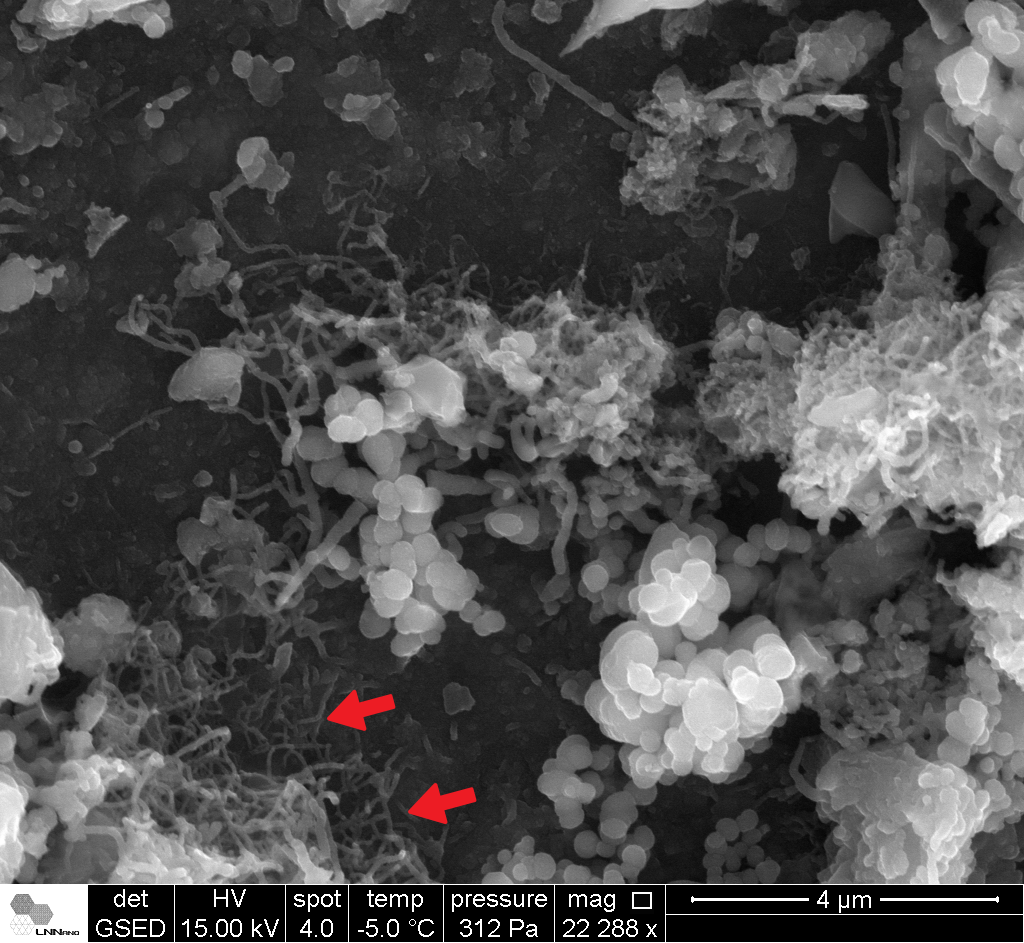


**Supplementary Table 3.** Mean coverage of MAGs and bins across samples.Mean coverage values of all recovered bins across three samples (FBA1, FBA2, FBA3). Coverage was calculated by mapping reads from each individual sample to the co-assembled contigs, followed by computing a length-weighted average coverage for each bin or MAG. Values are presented in base pairs mapped and reflect the relative abundance of each bin/MAG in the respective samples.

| **bins** | **FBA1** | **FBA2** | **FBA3** |
| --- | --- | --- | --- |
| **DI_Bin_00006** | 130.606.388.172 | 163.802.722.777 | 0.26098803798 |
| **DI_Bin_00007** | 0.0337329768683 | 369.504.530.645 | 0.12982457815 |
| **DI_Bin_00008** | 458.755.741.495 | 0.0855797659645 | 0.0358695371296 |
| **DI_Bin_00009** | 638.634.821.421 | 0.166532633149 | 0.0519889997687 |
| **DI_Bin_00010** | 408.694.137.567 | 630.687.582.412 | 216.724.543.952 |
| **DI_Bin_00011** | 525.086.766.139 | 183.543.489.841 | 131.741.160.912 |
| **DI_Bin_00012** | 0.0054282343993 | 152.271.210.766 | 0.181257678224 |
| **DI_Bin_00013** | 0.00244863078417 | 409.672.592.354 | 0.00701431248802 |
| **DI_Bin_00014** | 723.923.943.773 | 105.891.134.533 | 0.461470003643 |
| **DI_Bin_00015** | 0.329028374749 | 100.944.768.819 | 0.310825798798 |
| **DI_Bin_00016** | 0.0 | 0.0 | 357.737.589.701 |
| **DI_Bin_00017** | 0.0113334289141 | 215.404.278.447 | 0.549026574174 |
| **DI_Bin_00018** | 186.430.578.317 | 1.973.327.571 | 126.551.885.931 |
| **DI_Bin_00019** | 0.200561826607 | 110.424.460.745 | 573.302.449.918 |
| **DI_Bin_00020** | 760.788.427.205 | 0.0 | 0.0 |
| **DI_Bin_00021** | 162.589.947.008 | 0.00837129849691 | 0.0133197891055 |
| **DI_Bin_00022** | 0.574942919708 | 494.331.255.432 | 869.043.693.189 |
| **DI_Bin_00023** | 0.00735964804483 | 0.0145114156007 | 428.726.013.898 |
| **DI_Bin_00024** | 109.797.865.564 | 0.366000456427 | 27.251.761.021 |
| **DI_Bin_00025** | 105.255.663.703 | 240.172.034.209 | 913.626.409.057 |
| **DI_Bin_00026** | 4.365.855.848 | 254.156.985.113 | 375.942.776.536 |
| **DI_Bin_00027** | 620.435.305.606 | 118.749.087.029 | 180.366.639.681 |
| **DI_Bin_00028** | 150.720.133.456 | 0.86231499191 | 0.541666644763 |
| **DI_Bin_00029** | 708.587.640.428 | 470.511.995.741 | 837.422.106.178 |
| **DI_Bin_00030** | 0.0189368268042 | 973.595.301.334 | 0.0614388783154 |
| **DI_Bin_00031** | 539.371.440.948 | 528.378.183.573 | 0.478228630278 |
| **DI_Bin_00032** | 253.300.970.874 | 119.898.313.832 | 0.955825242718 |
| **DI_Bin_00033** | 0.0 | 0.0 | 859.559.471.366 |
| **DI_Bin_00034** | 179.435.078.477 | 0.0 | 0.0 |
| **DI_Bin_00035** | 59.264.091.858 | 0.0 | 0.616910229645 |
| **DI_MAG_00001** | 62.930.711.876 | 158.772.583.391 | 377.762.748.192 |
| **DI_MAG_00002** | 0.941655591365 | 434.631.495.605 | 373.845.788.138 |
| **DI_MAG_00003** | 0.16536614398 | 746.255.447.704 | 0.00218719295217 |
| **DI_MAG_00004** | 0.00131872760982 | 384.552.976.134 | 0.00628848755178 |
| **DI_MAG_00005** | 125.179.296.714 | 260.682.438.625 | 0.0265707653297 |

**Supplementary Table 4.** Percent recruitment of bins and MAGs across samples. This table shows the percent recruitment of metagenomic reads from each sample (FBA1, FBA2, FBA3) to the co-assembled bins. Percent recruitment indicates the proportion of reads from a given sample that map to each bin or MAG, reflecting its relative abundance and prevalence in the microbial community. The values provide insight into the distribution and representation of each bin/MAG across the different samples, with higher percentages indicating greater recruitment of reads. Unbinned reads are included under __splits_not_binned__.

| **samples** | **FBA1** | **FBA2** | **FBA3** |
| --- | --- | --- | --- |
| **DI_Bin_00006** | 0.5374119742607649 | 0.16566073342109727 | 0.022988930112228034 |
| **DI_Bin_00007** | 0.0035946313251844103 | 0.9677763344789401 | 0.029615007319366997 |
| **DI_Bin_00008** | 0.44690821649699114 | 0.020491014710556515 | 0.007480279701688168 |
| **DI_Bin_00009** | 0.15272796495454843 | 0.009788606341187495 | 0.0026615379954764576 |
| **DI_Bin_00010** | 0.6899167723822022 | 26.167.854.672.500.900 | 7.831.800.765.721.770 |
| **DI_Bin_00011** | 0.2234464713725672 | 0.19197158775769302 | 12.001.047.431.548.000 |
| **DI_Bin_00012** | 0.00012981490599870592 | 0.8950335505412579 | 0.009279351395726589 |
| **DI_Bin_00013** | 0.002678186100554831 | 11.013.108.062.681.600 | 0.016423200486767035 |
| **DI_Bin_00014** | 4.216.094.447.315.720 | 15.157.673.060.507.800 | 0.5753287866645329 |
| **DI_Bin_00015** | 0.10460654069191304 | 78.879.627.274.871.900 | 0.21154257409353044 |
| **DI_Bin_00016** | 0.0 | 0.0 | 0.029625763900264227 |
| **DI_Bin_00017** | 0.00031886611435904253 | 1.489.556.269.331.740 | 0.033067064960379436 |
| **DI_Bin_00018** | 0.5763191882954328 | 1.499.341.840.158.800 | 0.8374710293155667 |
| **DI_Bin_00019** | 0.00860528314124324 | 0.11644952444237223 | 0.5265697713702325 |
| **DI_Bin_00020** | 0.18729189995104975 | 0.0 | 0.0 |
| **DI_Bin_00021** | 1.155.625.351.607.280 | 0.014624187378093081 | 0.020266369563271873 |
| **DI_Bin_00022** | 0.00707701037140699 | 0.1495542353475793 | 0.22899286579990674 |
| **DI_Bin_00023** | 0.005383657692232469 | 0.0260906991383154 | 6.713.600.071.189.110 |
| **DI_Bin_00024** | 0.6603087702819668 | 0.05409913721490747 | 0.350835052515337 |
| **DI_Bin_00025** | 31.834.716.227.606.200 | 17.853.916.087.160.000 | 0.5915335637993294 |
| **DI_Bin_00026** | 19.960.425.821.392.500 | 2.855.998.690.514.070 | 36.794.032.924.016.500 |
| **DI_Bin_00027** | 0.0698238094082249 | 0.032846784351269996 | 0.04345287220376096 |
| **DI_Bin_00028** | 0.1166140848511494 | 0.0163983903054511 | 0.00897154148313898 |
| **DI_Bin_00029** | 0.027412163634314677 | 0.044737940439092655 | 0.06935046893794562 |
| **DI_Bin_00030** | 0.0005461074700146045 | 0.6900887114388886 | 0.003792882827194893 |
| **DI_Bin_00031** | 0.009484512414279665 | 0.02283639169086182 | 0.0018001880427094686 |
| **DI_Bin_00032** | 0.0035633124343095973 | 0.041455835125098366 | 0.002878397592985849 |
| **DI_Bin_00033** | 0.0 | 0.0 | 0.006471250713075812 |
| **DI_Bin_00034** | 0.1262101846829985 | 0.0 | 0.0 |
| **DI_Bin_00035** | 0.0020842446309753335 | 0.0 | 0.00046444497402279054 |
| **DI_MAG_00001** | 19.254.797.464.655.100 | 11.940.083.541.046.700 | 2.474.290.442.363.010 |
| **DI_MAG_00002** | 0.031461016638381724 | 0.3569089191882997 | 26.737.967.627.353.800 |
| **DI_MAG_00003** | 0.027450209027218127 | 3.044.683.226.874.630 | 0.0007772153908598205 |
| **DI_MAG_00004** | 0.000582044088109252 | 417.169.457.055.577 | 0.00594158380439406 |
| **DI_MAG_00005** | 163.769.304.536.494 | 0.838237945202044 | 0.007441479666155788 |
| **__splits_not_binned__** | 0.8145374288832262 | 6.414.581.522.207.170 | 16.398.736.834.921.900 |
